# Risk Assessment Scale for the Development of Medical Adhesive-related Skin Injuries*

**DOI:** 10.1590/1518-8345.7891.4710

**Published:** 2025-11-17

**Authors:** Maíla Fidalgo de Faria, Talita Silva Alves Tibola, Maria Beatriz Guimarães Raponi, Patrícia da Silva Pires, Maria Sagrario Gómez Cantarino, Maria Helena Barbosa

**Affiliations:** 1 Universidade Federal do Triângulo Mineiro, Uberaba, MG, Brazil.; 2 Scholarship holder at the Coordenação de Aperfeiçoamento de Pessoal de Nível Superior (CAPES), Brazil.; 3 Universidade Federal de Uberlândia, Faculdade de Medicina, Escola de Enfermagem, Uberlândia, MG, Brazil.; 4 Universidade Federal da Bahia, Escola de Enfermagem, Vitória da Conquista, BA, Brazil.; 5 Universidade de Castilla-La Mancha, Escola de Fisioterapia e Enfermagem, Toledo, Castilla-La Mancha, Spain.; 6 Scholarship holder at the Conselho Nacional de Desenvolvimento Científico e Tecnológico (CNPq), Brazil.

**Keywords:** Adhesives, Wounds and Injuries, Skin, Patient Safety, Evidence-Based Nursing, Secondary Prevention

## Abstract

to develop and validate the Risk Assessment Scale for the Development of Medical Adhesive-related Skin Injuries in hospitalized adults.

methodological study consisting of the development, face validity, content validity, predictive criterion validity, and interobserver reliability analysis of the scale. It was developed using risk factors obtained in an integrative review and underwent face and content validation by seven judges. Three hundred and twenty-nine patients participated in the predictive criterion validity and 32 in the reliability analysis.

the scale consists of 48 items and eight domains. Scores < 11 classify patients as low risk and ≥ 11 as high risk for developing medical adhesive injuries. In face validity, there was 100% agreement among the judges, and in content validity, a total content validity coefficient of 0.94 was obtained. In predictive criterion validity, there was a statistically significant association (p < 0.001) between the score generated by the scale and the occurrence of medical adhesive injuries. The reliability analysis obtained an intraclass correlation coefficient of 0.92.

the scale developed is a valid and reliable tool for identifying the risk of injury from medical adhesives in hospitalized adults.

## Highlights

**(1)** ERLAM is an instrument with excellent accuracy, sensitivity, and specificity. **(2)** ERLAM is a valid and reliable instrument for identifying the risk of MARSI. **(3)** ERLAM will assist in the prevention of MARSI in hospitalized adults. **(4)** ERLAM is validated and ready for use in Brazilian Portuguese.

## Introduction

Medical adhesive-related skin injuries (MARSI) are characterized by abnormalities present on the skin 30 minutes or more after removal of the adhesive^(1)^. These are preventable adverse events that occur in any healthcare setting and compromise patient comfort and safety as well as the quality of nursing care. In addition, these injuries are iatrogenic acts that can trigger ethical and legal implications for healthcare professionals and institutions^(2)^.

MARSI have several etiologies and are classified into mechanical injuries (abrasions, blisters, and friction injuries), dermatitis (irritant and allergic contact), and other causes (maceration and folliculitis)^(1)^. The incidence of these injuries varies according to the population evaluated, with reports in the literature ranging from 0.1% to 60.3%^(3)^.

Identifying risk factors is essential for the prevention of MARSI. However, due to the absence of validated instruments for predicting the occurrence of these lesions, some studies have used the Braden Scale to predict the occurrence of MARSI^(2)^. However, there are limitations to this type of study, since it is not a specific instrument for this assessment. It should be noted that risk classifications should be performed using validated instruments that ensure adequate levels of specificity and sensitivity. The updated expert consensus on MARSI considers the assessment of the patient’s risk of developing MARSI as one of the main pillars for the prevention of these injuries^(4)^. Therefore, it is necessary to develop and validate an instrument capable of classifying patients according to their risk of developing these injuries during hospitalization.

Factors inherent to the patient or the external environment can increase the predisposition to MARSI during healthcare. Intrinsic risk factors include: age > 50 years; low serum albumin level; skin edema at the adhesive fixation site; history of MARSI; dry skin; history of skin allergies; high skin moisture; diabetes mellitus; low body weight; hypoproteinemia; presence of comorbidities; skin problems; history of food allergies; decreased skin elasticity; fever. Extrinsic risk factors include: history of smoking; use of sedatives; high frequency of adhesive use; type of adhesive used; use of immunosuppressants; use of anticoagulants; use of mechanical ventilation; high Braden score^(5)^.

Given the need to identify risk factors to implement measures to prevent MARSI, the overall objective of this study was to develop and validate the Risk Assessment Scale for the Development of Medical Adhesive-Related Skin Injuries in hospitalized adults. The specific objectives were: to conduct an integrative review to gather scientific evidence for the development of the scale; to perform apparent and content validation of the developed scale; to evaluate the predictive criterion validity of the developed scale; to evaluate the interobserver reliability of the developed scale.

## Method

### Study design

This is a methodological study on the construction and validation of a scale conducted in three stages:

- Stage 1 - Review study: integrative review of risk factors for MARSI in hospitalized adults;
- Stage 2 - Methodological study: development of ERLAM based on the results of the integrative review and face and content validation of the scale developed;
- Stage 3- Observational study: analysis of metric properties (predictive criterion validity and interobserver reliability) of the final version of ERLAM.

### Step 1 – Review study (integrative review)

#### Data collection location

To construct the items on the scale, risk factors obtained through an integrative review registered in the Open Science Framework, DOI: https://doi.org/10.17605/OSF.IO/346YF, were used. The review was conducted in the PubMed, Cochrane Library, Embase, LILACS, CINAHL, and Google Scholar databases.

#### Period

The articles were searched for in October 2021.

#### Selection criteria

The integrative review was conducted according to the steps recommended in the literature^(6)^ and in the PRISMA guideline^(7)^. To meet the objective of the review, the PICo mnemonic was structured as follows:

- Population (P) = hospitalized adults;
- Interest (I) = risk factors for MARSI occurrence;
- Context (Co) = use of medical adhesives in healthcare.

Based on the mnemonic developed, the following review question was formulated: “What are the risk factors for MARSI in hospitalized adults who use medical adhesives during healthcare?” Primary studies that answered the review question, in English, Portuguese, or Spanish, with no publication year restrictions, were adopted as inclusion criteria. Articles that exclusively addressed children, event abstracts, book chapters, letters, manuals, editorials, review methods, case reports, duplicate articles, and those that did not address the topic under study were excluded. PRISMA diagram is available from https://doi.org/10.48331/SCIELODATA.RCBYDO.

#### Tools used to collect information

For data extraction and evidence synthesis, a table was created containing: author(s); year of publication; objectives; population and sample size; method; type and duration of intervention (if any); main findings related to risk factors for MARSI in hospitalized adults; level of evidence. The classification of the level of evidence followed the criteria of the Oxford Centre for Evidence-Based Medicine, which uses a classification from 1 to 5, with 1 being the highest level of evidence and 5 the lowest^(8)^.

#### Data collection

The articles identified in the search were exported to the free Rayyan QCRI application and selected through analysis of the title and abstract, individually by two researchers, in a blind manner, according to the inclusion and exclusion criteria adopted.

After this selection, a consensus meeting was held between the researchers and a third evaluator, resulting in a list of articles that were read in full. The reference lists of the selected articles were also consulted, and those that met the inclusion criteria were added to the final sample of articles.

#### Data processing and analysis

The risk factors identified in the integrative review were grouped into categories. Risk factors were variables presented in the studies as significant predictors for the occurrence of MARSI (p < 0.05), factors common to more than one study, as well as the criteria established in the consensus developed by McNichol, et al.^(1)^ for the assessment, prevention, and treatment of MARSI.

#### Ethical aspects

This study followed all ethical precepts regarding the copyright of articles included in the review stage.

### Stage 2 – Methodological study: face and content validation

#### Data collection location

The face and content validation stages of ERLAM took place online through forms sent to the emails of the selected judges.

#### Period

After being developed, ERLAM underwent rounds of content validation from June 21, 2022, to December 19, 2022.

#### Population

Professionals who met the following inclusion criteria were invited to participate as judges in the scale validation process: nurses, physicians, working in hospitals and scientific production related to skin lesions.

#### Selection criteria

The search for professionals who met the inclusion criteria was conducted on the Lattes Platform, using the keyword “Skin” in the subject search. The filter “professional activity” was used with the broad area “Health Sciences,” the area “nursing,” and the subareas “adult and elderly health nursing.” A second search was also performed using the keyword “wounds.” Emails were sent inviting all 37 professionals identified in the search who met the inclusion criteria to participate in the validation of the scale. However, after three attempts to contact them, only seven agreed to participate in the apparent and content validation stages of ERLAM.

#### Study variables

In terms of face validity, the judges assessed the suitability of the content for the audience that will use the scale, whether the structure and content of the domain were correct, and whether the content was representative^(9)^. In terms of content validity, the ERLAM items were evaluated according to the following criteria:

- Clarity of language: how clear, understandable, and appropriate is the language of the item for healthcare professionals?
- Practical relevance: how important is the item for the instrument?
- Theoretical relevance: what is the association between the item and the construct (risk of medical adhesive-related skin injury)?

#### Tools used to collect information

Online forms containing all items on the scale were used in the validation rounds. In face validity, the options “agree” or “disagree” should be marked for each item. When there was disagreement with any of the items proposed on the scale (ERLAM), the justification was reported in a specific space by the judge. The responses were compared to verify agreement among judges^(10)^. In content validity, the items should be scored from 1 to 5, where 1 = very little, 2 = little, 3 = average, 4 = a lot, and 5 = very much. Items scored from 1 to 3 should be justified by the judges in the “observation” space.

#### Data collection

Validation rounds were conducted, in which the instruments were sent to the judges and returned to the researchers. The scale was modified according to the judges’ suggestions and submitted to a new validation round. This process was repeated until the appropriate agreement and content validity coefficient (CVC) indices were achieved.

#### Data processing and analysis

In terms of face validity, items that obtained an agreement index equal to or greater than 80% were accepted^(11)^. Items with less than 80% were adjusted according to suggestions and sent to the judges for another round of analysis. Those that remained below the recommended index after adjustments were excluded or readjusted, generating new rounds of analysis. In content validity, the CVC was calculated to identify items that did not meet the objectives of the instrument^(12)^.

#### Ethical aspects

This study followed ethical guidelines and regulatory standards for research conducted in a virtual environment. The research was only conducted after approval by the Research Ethics Committee (No. 4,788,062 and CAAE: 44205421.3.0000.8667). All participating judges signed a free and informed consent form.

### Step 3 - Observational study: assessment of interobserver reliability and predictive criterion validity

#### Data collection site

To assess interobserver reliability and the predictive validity of ERLAM, an observational, longitudinal study was conducted in three intensive care units (two general adult units and one coronary care unit) and a surgical clinic unit at a public teaching hospital in Uberaba, Minas Gerais, Brazil.

#### Period

Before starting the observational study for predictive criterion validity and interobserver reliability, a pretest was conducted with 32 patients from February 14, 2023, to March 9, 2023 (not included in the study sample). The observational study took place from March 9, 2023, to October 28, 2023.

#### Population

The validity of predictive criteria and interobserver reliability was assessed in all patients admitted to the: Adult Intensive Care Unit (ICU-A), Coronary Intensive Care Unit (ICU-C), Type II Intensive Care Unit (ICU-II) and Surgical Clinic (CC) units during the data collection period who met the inclusion criteria.

#### Selection criteria

All patients aged 18 years or older who were using at least one medical adhesive patch affixed to the skin were included. Those with previous skin integrity changes that compromised the assessment at the patch application site were excluded.

#### Study variables

For predictive and interobserver validity, ERLAM scale scores were recorded, as well as data regarding participant characteristics, medical adhesives used, and the appearance of lesions.

#### Tools used to collect information

To record the variables analyzed in the study, a data collection instrument developed by the researchers was used, consisting of the patient’s sociodemographic characteristics (gender, age, hospitalization sector, date of admission, medical diagnosis, dates of the first and last day of data collection, and reason for ending data collection), medical adhesives in use (type of adhesive, location of application, date of insertion, and date of removal), ERLAM scale, and data on MARSI (whether there is an injury, date on which the injury was identified, type of injury, location of the injury, and medical adhesive predictive of the injury). The instrument is available from https://doi.org/10.48331/SCIELODATA.RCBYDO.

#### Data collection

The criteria for identifying and classifying MARSI are not yet widely known among nursing professionals in clinical practice^(13)^. Therefore, to avoid the risk of bias in the process of identifying the emergence of MARSI in the patients evaluated in the study, the steps of predictive criterion validity and interobserver reliability were performed by two nurses (one master’s student and one doctoral student) belonging to the Study and Research Group on Evidence-Based Practice and Patient Safety in the Care Process, who underwent training to assess the onset of MARSI according to the criteria recommended by McNichol and colleagues^(1)^.

The training took place before the start of the research and lasted six hours a day for three days, until there was standardization in the assessments of the patients’ skin, leveling their ability to identify the presence of MARSI. It should be noted that both are also professionals working in clinical nursing practice.

In the interobserver analysis, the researchers evaluated the patients’ body surface in the areas where the adhesive patches were affixed and applied the scale (ERLAM) simultaneously without communicating with each other. The assessments took place during the change or removal of the adhesive pads after bathing. This procedure was performed once a day until one of the following outcomes was achieved: patient discharge from the ward, bed transfer, death, absence of adhesive pads, completion of seven days of follow-up, or the appearance of MARSI. The patients’ skin was not assessed during adhesive changes that occurred outside bathing times due to the impossibility of the researchers remaining in the ward full-time. At this stage, the risk of bias was minimized, as the researchers did not have access to the cut-off points for high or low risk of MARSI proposed by ERLAM.

#### Data processing and analysis

For predictive criterion validity, the sample size calculation considered a lesion prevalence of 30%, accuracy of 5%, confidence interval of 95%, and sample loss of 20%, which generated a minimum sample of 322 participants and a maximum of 414. The sample size calculation for interobserver reliability analysis considered an expected intraclass correlation coefficient (ICC) of 0.9 between ERLAM scores, assuming that this is not less than 0.75% for a power of 80% with a significance level of α = 0.05^(14)^. Entering these values into the Power Analysis and Sample Size (PASS) application, version 15, a minimum sample size of n = 27 patients was obtained.

Interobserver reliability was analyzed using the Kappa coefficient^(15)^. Predictive criterion validity was analyzed using univariate logistic regression. The cut-off point for the best sensitivity and specificity values of the scale was determined using Receiver Operating Characteristic (ROC) curve (available from https://doi.org/10.48331/SCIELODATA.RCBYDO) analysis. The ICC was used for the total risk score for the development of lesions associated with the use of medical adhesives^(14)^. Thus, it was possible to measure the accuracy of ERLAM in predicting the occurrence of MARSI.

#### Ethical aspects

This research followed ethical guidelines and regulatory standards for research involving human subjects and was conducted only after approval by the Research Ethics Committee (No. 4,788,062 and CAAE: 44205421.3.0000.8667). The patients who participated in the predictive criterion validity and interobserver reliability stages, or their legal representatives, signed the informed consent form.

## Results

The integrative review resulted in the inclusion of 14 articles that addressed risk factors predictive of MARSI in adults. Forty-one risk factors were selected and grouped into eight domains, which comprised the initial version of ERLAM (Domain 1—Epidemiological and clinical aspects; Domain 2—Characteristics of the skin at the adhesive attachment site; Domain 3 - Medications in use; Domain 4 - Medical devices in use; Domain 5 - Types of adhesives in use; Domain 6 - Adhesive attachment site; Domain 7 - Adhesive use; Domain 8 - Hospitalization). This version was sent to the judges for face and content validation.

The first round of face validation involved seven judges. Of these, four judges withdrew from the validation rounds, leaving three judges in the final round of content validation. After three rounds of face validation and modification of items as suggested by the judges (Figure 1), there was 100% agreement among the judges regarding the items on the scale. The reduction in the number of judges did not interfere with the validation result, since the final number of judges (three) still remained within the minimum recommended by the literature^(12)^.

**Figure 1-:** Suggested modifications to the items on the Risk Assessment Scale for the Development of Medical Adhesive-related Skin Injuries in the face validation rounds. Uberaba, MG, Brazil, 2022

**Item (% agreement among judges)**	**Suggested modification**	**Final version of the item**
**First round of face validation (7 judges)**		
Cancer patient (57.1%)	Patient undergoing radiation and/or chemotherapy	Cancer patient*
	Cancer patient	
Postoperative hip surgery (57.1%)	Postoperative care for major surgery	Postoperative care for major surgery
**Second round of face validation (5 judges)**		
Postoperative care for major surgery (60%)	Some major surgeries, such as orthopedic surgery, do not use adhesives to secure dressings.	Post-operative care for major surgery*
Adhesives in recurrent sites (60%)	Frequent removal	Adhesives in recurrent sites*
**Third round of face validation (3 judges)**		
Body temperature ≥ 37.5°C (66.6%)	In a feverish state, it is probably more difficult.	Body temperature ≥ 37.5°C*
Enteral/nasogastric tube (66.6%)	Replace the term catheter with probe.	Enteral/nasogastric tube*
Indwelling urinary catheter (66.6%)	Replace the term catheter with probe.	Indwelling urinary catheter*
Acrylate tape (66.6%)	Add an example to the item.	Acrylate adhesive tape (e.g., Medipore ^®^ , Durapore ^®^ )
Upper limb (66.6%)	Subdivide the upper limb into parts, as some areas are more vulnerable to injury than others.	Upper limb*

*After contacting the judges, it was agreed that the term would be maintained

In the first round of content validation, a total CVC of 0.94 and a final CVC of each item ≥ 0.88 were obtained for all aspects evaluated (clarity, theoretical relevance, and practical relevance), which corresponds to the full approval of the items. Therefore, for 94% of the evaluators, ERLAM was considered relevant for predicting the risk of developing MARSI in hospitalized adults.

**Figure 2–:**
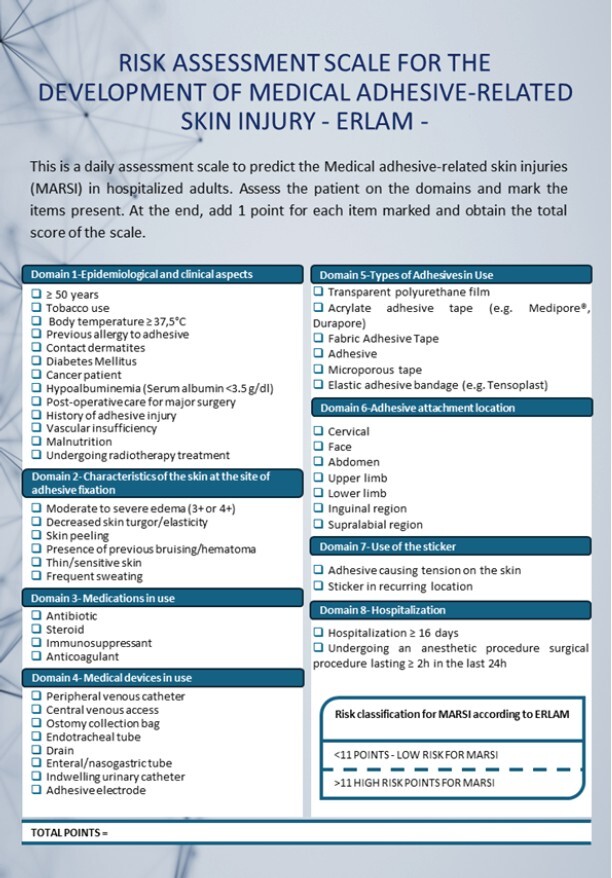
Final version of the Risk Assessment Scale for the Development of Medical Adhesive-related Skin Injuries

Thus, the final version of ERLAM was obtained, consisting of 48 items and eight domains (Figure 2).

To obtain the total score on the scale, one point must be added for each item identified in the patient. Thus, the ERLAM score can range from zero to 48 points. To classify MARSI risk as high or low, the score adopted by the ROC curve was 11 points, as this value presented the best relationship between the sensitivity and specificity of the scale. Thus, scores < 11 classify patients as low risk and scores ≥ 11 as high risk for developing MARSI.

To analyze the predictive validity of ERLAM, 329 patients were evaluated, and 32 were evaluated for interobserver reliability. Among the participants, 193 (58.7%) were male, the minimum age was 19 years, and the maximum age was 91 years, with a mean age of 57.00. Patients aged 60 years or older represented 54.5% of the sample. One hundred and ten (33.4%) were admitted to the surgical clinic, 110 (33.4%) to the ICU-C or ICU-II, and 109 (33.2%) to ICU-A.

The ERLAM scores obtained during the observational study of the predictive criterion validity stage ranged from four to 27 points. In total, 143 (43.5%) patients developed some type of MARSI. Logistic regression of the association between ERLAM scores and the occurrence of MARSI found that the mean score was 15.07 points among patients who developed injury and 10.9 points among the 186 (56.5%) patients who did not develop injury (Table 1).

**Table 1–:** Logistic regression of the association between scores on the Risk Assessment Scale for the Development of Medical Adhesive-related Skin Injuries and its occurrence. Uberaba, MG, Brazil, 2023

Occurrence of MARSI*	**n** ^†^ **(%)**	**Min.-max.**	**Mean**	**Standard deviation**	**Median**	**OR** ^‡^	**95%CI** ^§^
Yes	143 (43.5)	4 - 24	15.07	4.11	15.00		
						1.27	1.19 -1.35
No	186 (56.5)	4 - 27	10.90	4.10	10.00		

*MARSI = Medical adhesive-related skin injuries; ^†^n = Number of participants; ^‡^OR = Odds ratio; ^§^CI = Confidence interval

ERLAM presented an odds ratio (OR) of 1.27, which demonstrates a statistically significant association (p < 0.001) between the score generated by the scale and the occurrence of MARSI. For each point recorded in the score, there is a 27.0% increase in the chance of MARSI occurring. Therefore, ERLAM has predictive criterion validity associated with the occurrence of MARSI in hospitalized adults. In addition, the analyses also demonstrated that patients classified as high risk for MARSI, according to the ERLAM classification, are 8.14 times more likely to develop MARSI during hospitalization than those classified as low risk (Table 1). The logistic regression between the ERLAM risk classification, with a cut-off point of 11 points, and the occurrence of MARSI showed a sensitivity of 86% and specificity of 57% (Table 2).

**Table 2-:** Relationship between the risk classification of the Risk Assessment Scale for the Development of Medical Adhesive-related Skin Injuries and its occurrence. Uberaba, MG, Brazil, 2023

	ERLAM*		
Presence of MARSI ^†^	High risk for MARSI ^†^ n ^‡^ (%)	Low risk for MARSI ^†^ n ^‡^ (%)	Total n ^‡^ (%)
Yes	123 (86.00%)	20 (14.00%)	143 (100.00%)
No	80 (43.00%)	106 (57.00%)	186 (100.00%)
Total n (%)	203 (61.70%)	126 (38.30%)	329 (100.00%)

*ERLAM = The Risk Assessment Scale for the Development of Medical Adhesive-related Skin Injuries; ^†^MARSI = Medical adhesive-related skin injury; ^‡^n = Number of participants

Interobserver reliability showed that 39 (81.5%) items on the scale had kappa values corresponding to the best agreement indices, with 30 (62.5%) items showing perfect agreement and nine (19.0%) showing substantial agreement. All ERLAM items reached significance levels of p < 0.05, with most being p = 0.001. The level of agreement between observers ranged from 75% to 100.0%. The interobserver reliability analysis of the ERLAM (Table 3) obtained an ICC of 0.92, classified as excellent^(13)^ and a p<0.05, which demonstrates the high interobserver reliability of the scale.

**Table 3-:** Interobserver reliability analysis of the Risk Assessment Scale for the Development of Medical Adhesive-related Skin Injuries. Uberaba, MG, Brazil, 2023

**Observer**	**Min.**	**Max.**	**Mean**	**Median**	**Standard Deviation**	ICC*	**p** ^†^
**Obs. 1**	9	22	14.38	14.0	3.36	0.92	< 0.05
**Obs. 2**	9	22	13.81	14.0	3.22		

*ICC = Intraclass correlation coefficient; ^†^p = Significance level

## Discussion

After searching the literature, no scale was identified that classifies the risk of developing MARSI in hospitalized adults. Regarding content validity, ERLAM presented significant values, similar to those of scales that assess the risk of skin lesions, such as the Risk Assessment Scale for the Development of Lesions Resulting from Surgical Patient Positioning (ELPO)^(16)^ and the Risk Assessment Scale for Adhesive-Related Skin Lesions at the PICC Insertion Site in Cancer Patients^(17)^. In these scales, the content validity index (CVI) ranged from 0.83^(17)^ to 0.88^(16)^.

In ERLAM, the difference between the scores of the groups classified as high or low risk for MARSI was more than four points in the total score, close to that presented by ELPO, which obtained a difference between groups with or without pressure injury (PI) of almost 5 points (p < 0.001)^(16)^. The analyses of the metric properties of ERLAM also showed excellent results for accuracy, sensitivity, specificity, and RC. These values were also close to those presented in the validation process of the other scales. ELPO^(16)^ showed an accuracy of 0.83, sensitivity of 88.0%, specificity of 64.4%, and RC of 1.44. The metric properties of Braden Scale^(18)^ varied according to the level of education of the professionals who used the scale. Therefore, although Braden’s sensitivity was 100%, specificity ranged from 64% to 90% among technicians and nurses, respectively. The scale for assessing the risk of skin injury related to the adhesive at the PICC insertion site in cancer patients^(17)^ had an accuracy of 0.75, sensitivity of 80.0%, and specificity of 65.5%, and the scale for assessing the risk of MARSI in infants and children in intensive care units^(19)^ had an accuracy of 0.92, sensitivity of 86.27%, and specificity of 98.76%.

It should be noted that the sensitivity and specificity values of an instrument may vary after validation, depending on the population in which it is applied. For example, the Braden scale, when applied in different studies after validation, presented sensitivity values ranging from 31.2% to 95% and specificity values ranging from 16.6% to 88.2%^(20)^.

The interobserver reliability of ERLAM achieved excellent results, as did the other instruments, adopting the reference value of ICC ≥0.75^(14)^. The reliability of Braden varied according to the level of education of the professional who used the scale (0.83 to 0.94 for nursing assistants and 0.99 for nurses)^(18)^. ELPO had an ICC of 0.99^(16)^, while the scale for assessing the risk of skin damage related to the adhesive at the PICC insertion site in cancer patients^(17)^ had an ICC of 0.97 and the scale for assessing the risk of MARSI in infants and children in intensive care units^(19)^ had an ICC of 0.85.

The absence of another risk assessment scale for MARSI in adults to perform concurrent validity was identified as a limitation of the study. ERLAM was created and validated in Brazilian Portuguese. Therefore, further studies are needed to translate and validate the use of the scale in other languages so that it can be used in other countries.

The ERLAM can assist in evidence-based nursing practice, as it objectively gathered the best scientific evidence on risk factors for MARSI in hospitalized adults. In addition, by classifying this population according to the risk of developing these injuries, the scale will allow nurses to identify patients at risk for MARSI more quickly and accurately.

## Conclusion

Through predictive criterion validity and interobserver reliability analyses, ERLAM demonstrated excellent accuracy, sensitivity, and specificity. Therefore, this is a valid and reliable tool for identifying the risk of MARSI in hospitalized adults.

## Data Availability

All data generated or analysed during this study are included in this published article.
