# Supplementary material for: Risk Assessment Scale for the Development of Medical Adhesive-related Skin Injuries
Source: Rev Lat Am Enfermagem. 2025 Nov 17;33:e4710. doi: 10.1590/1518-8345.7891.4710 (PMC12628365; doi:10.1590/1518-8345.7891.4710)
Supplement: Translation

# Translation: Escala de Avaliação de Risco para o Desenvolvimento de Lesões de pele Relacionadas a Adesivos Médicos*

**DOI:** 10.1590/1518-8345.7891.4711

**Authors:** Maíla Fidalgo de Faria, Talita Silva Alves Tibola, Maria Beatriz Guimarães Raponi, Patrícia da Silva Pires, Maria Sagrario Gómez Cantarino, Maria Helena Barbosa

**Affiliations:** 1 Universidade Federal do Triângulo Mineiro, Uberaba, MG, Brasil.; 2 Bolsista da Coordenação de Aperfeiçoamento de Pessoal de Nível Superior (CAPES), Brasil.; 3 Universidade Federal de Uberlândia, Faculdade de Medicina, Escola de Enfermagem, Uberlândia, MG, Brasil.; 4 Universidade Federal da Bahia, Escola de Enfermagem, Vitória da Conquista, BA, Brasil.; 5 Universidade de Castilla-La Mancha, Escola de Fisioterapia e Enfermagem, Toledo, Castilla-La Mancha, Espanha.; 6 Bolsista do Conselho Nacional de Desenvolvimento Científico e Tecnológico (CNPq), Brasil.

**Keywords:** Adesivos, Ferimentos e Lesões, Pele, Segurança do Paciente, Enfermagem Baseada em Evidências, Prevenção Secundária

## Abstract

desenvolver e validar a Escala de Avaliação de Risco para o Desenvolvimento de Lesões de pele Relacionadas a Adesivos Médicos em adultos hospitalizados.

estudo metodológico composto por elaboração, validação de face, de conteúdo, de critério preditiva e análise de confiabilidade interobservadores da escala. Ela foi elaborada utilizando fatores de risco obtidos em revisão integrativa e passou por validação de face e conteúdo por sete juízes. Trezentos e vinte nove pacientes participaram da validade de critério preditiva e 32 da confiabilidade.

a escala é composta por 48 itens e oito domínios. Os escores < 11 classificam os pacientes em baixo risco e os ≥ 11 para alto risco de desenvolver lesões por adesivos médicos. Na validade de face, houve concordância de 100% dos juízes, na de conteúdo obteve-se coeficiente de validade de conteúdo total de 0,94. Na validade de critério preditiva houve associação estatisticamente significativa (p < 0,001) entre o escore gerado pela escala e a ocorrência de lesões por adesivos médicos. A análise de confiabilidade obteve um coeficiente de correlação intraclasse de 0,92.

a escala desenvolvida é um instrumento válido e confiável para identificar o risco da ocorrência de lesões por adesivos médicos em adultos hospitalizados.

## Destaques

**(1)** A ERLAM é um instrumento que possui excelente acurácia, sensibilidade e especificidade. **(2)** A ERLAM é um instrumento válido e confiável para identificar o risco de MARSI. **(3)** A ERLAM atuará no auxílio à prevenção de MARSI em adultos hospitalizados. **(4)** A ERLAM está validada e pronta para uso no português do Brasil.

## Introdução

As lesões de pele relacionadas a adesivos médicos (*medical adhesive-related skin injury* - MARSI) são caracterizadas por anormalidades presentes na pele após 30 minutos ou mais da retirada do adesivo^(1)^. Estes são eventos adversos evitáveis que estão presentes em qualquer cenário da assistência e comprometem o conforto e segurança do paciente assim como a qualidade da assistência de enfermagem. Além disso, as lesões são atos iatrogênicos que podem desencadear implicações éticas e legais para os profissionais e instituições de saúde^(2)^.

As MARSI possuem diversas etiologias e são classificadas em lesões mecânicas (desnudamento, bolhas e lesões por fricção), dermatites (de contato irritante e alérgica) e outras causas (maceração e foliculites)^(1)^. A incidência dessas lesões sofre alterações de acordo com a população avaliada, com registros na literatura que vão de 0,1% a 60,3%^(3)^.

A identificação dos fatores de risco é fundamental para a prevenção das MARSI. Porém, devido à ausência de instrumentos validados para a predição da ocorrência dessas lesões, alguns estudos utilizaram a Escala de Braden na tentativa de predizer a ocorrência de MARSI^(2)^. Entretanto, há limitações nesse tipo de estudo, uma vez que não se trata de um instrumento específico para esta avaliação. Ressalta-se que classificações de risco devem ser realizadas por meio de instrumentos validados, que garantam níveis adequados de especificidade e sensibilidade. A atualização do consenso de especialistas sobre MARSI traz a avaliação do paciente quanto o risco de desenvolvimento de MARSI como um dos principais pilares para a prevenção dessas lesões^(4)^. Portanto, faz-se necessária a elaboração e validação de um instrumento capaz de classificar os pacientes quanto ao risco de desenvolverem essas lesões durante a hospitalização.

Fatores inerentes ao paciente ou ao meio externo podem aumentar a predisposição à ocorrência de MARSI durante a assistência à saúde. Quanto aos fatores de risco intrínsecos, destacam-se: idade > 50 anos; baixo nível de albumina sérica; edema de pele no local de fixação do adesivo; histórico de MARSI; pele seca; histórico de alergias de pele; alta umidade da pele; Diabetes mellitus; baixo peso corporal; hipoproteinemia; presença de comorbidades; problemas de pele; histórico de alergias alimentares; elasticidade da pele diminuída; febre. Dentre os fatores de risco extrínsecos estão: histórico de tabagismo; uso de sedativo; alta frequência de uso de adesivos; tipo de adesivo em uso; uso de imunossupressor; uso de anticoagulante; uso de ventilação mecânica; escore de Braden elevado^(5)^.

Tendo em vista a necessidade de identificar fatores de risco para implementar medidas que previnam as MARSI, este estudo teve como objetivo geral desenvolver e validar a Escala de Avaliação de Risco para o Desenvolvimento de Lesões de pele Relacionadas a Adesivos Médicos em adultos hospitalizados. Os objetivos específicos foram: realizar revisão integrativa para reunir evidências científicas para o desenvolvimento da escala; realizar validação aparente e de conteúdo da escala elaborada; avaliar a validade de critério preditiva da escala elaborada; avaliar a confiabilidade interobsevadores da escala elaborada.

## Método

### Delineamento do estudo

Trata-se de estudo metodológico de construção e validação de uma escala conduzido em três etapas:

- Etapa 1- Estudo de revisão: revisão integrativa dos fatores de risco para MARSI em adultos hospitalizados;
- Etapa 2- Estudo metodológico: desenvolvimento da ERLAM a partir dos resultados da revisão integrativa e validação de face e conteúdo da escala elaborada;
- Etapa 3- Estudo observacional: análise de propriedades métricas (validade de critério preditiva e confiabilidade interobservadores) da versão final da ERLAM.

### Etapa 1- Estudo de revisão (revisão integrativa)

#### Local da coleta de dados

Para construção dos itens da escala utilizou-se os fatores de risco obtidos por meio de revisão integrativa protocolada no *Open Science Framework*, DOI: https://doi.org/10.17605/OSF.IO/346YF. A revisão ocorreu nas bases de dados *PubMed*, *Cochrane Library*, *Embase*, Lilacs, CINAHL e buscador *Google Scholar*.

#### Período

As buscas dos artigos foram realizadas em outubro de 2021.

#### Critérios de seleção

A revisão integrativa foi conduzida conforme etapas preconizadas na literatura^(6)^ e no *guideline* PRISMA^(7)^. Para atender ao objetivo da revisão, o mnemônico PICo ficou estruturado da seguinte forma:

- População (P) = adultos hospitalizados;
- Interesse (I) = fatores de risco para ocorrência de MARSI;
- Contexto (Co) = utilização de adesivos médicos na assistência à saúde.

A partir do mnemônico elaborado, chegou-se à seguinte questão de revisão: “Quais os fatores de risco para a ocorrência de MARSI em adultos hospitalizados que utilizam adesivos médicos durante a assistência à saúde?”. Foram adotados como critérios de inclusão estudos primários que respondiam à questão de revisão, em inglês, português ou espanhol, sem delimitação de ano de publicação. Foram excluídos artigos que abordavam exclusivamente crianças, resumos de eventos, capítulos de livros, cartas, manuais, editoriais, métodos de revisão, relatos de caso, artigos duplicados e os que não contemplavam o tema em estudo. O diagrama PRISMA referente à revisão encontra-se depositado como material suplementar em https://doi.org/10.48331/SCIELODATA.RCBYDO.

#### Instrumentos utilizados para a coleta das informações

Para extração de dados e síntese de evidências foi elaborada uma tabela contendo: autor/autores; ano de publicação; objetivos; população e tamanho amostral; método; tipo e duração da intervenção (se houver); principais achados relacionados aos fatores de risco para MARSI em adultos hospitalizados; nível de evidência. A classificação do nível de evidência seguiu os critérios do *Oxford Centre Evidence-Based Medicine*, que utiliza classificação de um (1) a cinco (5), sendo 1 o maior nível de evidência e 5 o menor^(8)^.

#### Coleta de dados

Os artigos identificados na busca foram exportados para o aplicativo gratuito *Rayyan* QCRI, e selecionados por meio da análise do título e resumo, individualmente por duas pesquisadoras, de forma cega, conforme critérios de inclusão e exclusão adotados.

Após a seleção, houve uma reunião de consenso entre as pesquisadoras e um terceiro avaliador, em que se obteve a lista de artigos que foram lidos na íntegra. As listas de referência dos artigos selecionados também foram consultadas e aqueles que contemplavam os critérios de inclusão foram adicionados à amostra final de artigos.

#### Tratamento e análise dos dados

Os fatores de risco identificados na revisão integrativa foram agrupados em categorias. Considerou-se fatores de risco as variáveis apresentadas nos estudos como preditores significativos para ocorrência de MARSI (p < 0,05), fatores em comum apresentados em mais de um estudo, assim como os critérios estabelecidos no consenso elaborado por McNichol e colaboradores^(1)^ para avaliação, prevenção e tratamento de MARSI.

#### Aspectos éticos

Este estudo seguiu todo os preceitos éticos acerca dos direitos autorais dos artigos incluídos na etapa de revisão.

### Etapa 2- Estudo metodológico: validação de face e conteúdo

#### Local da coleta de dados

As etapas de validação de face e conteúdo da ERLAM ocorreram de forma *online* por meio de formulários enviados aos *e-mails* dos juízes selecionados.

#### Período

Após elaborada, a ERLAM passou por rodadas de validação de face de conteúdo, no período de 21/06/2022 a 19/12/2022.

#### População

Foram convidados a participar como juízes no processo de validação da escala os profissionais que atendessem aos seguintes critérios de inclusão: serem enfermeiros, doutores, com atuação em área hospitalar e produção científica relacionada a lesões de pele.

#### Critérios de seleção

A busca dos profissionais que atendiam aos critérios de inclusão foi feita na Plataforma Lattes, com uso da palavra-chave “Pele” na busca por assunto. Utilizou-se o filtro “atuação profissional” com grande área “Ciências da Saúde”, área “enfermagem” e subáreas “enfermagem em saúde do adulto e idoso”. Também foi feita uma segunda busca com a palavra-chave “feridas”. Foram enviados e-mails com convite para participar da validação da escala para todos os 37 profissionais identificados na busca, que atendiam aos critérios de inclusão. Porém, após três tentativas de contato, somente sete aceitaram participar das etapas de validação aparente e de conteúdo da ERLAM.

#### Variáveis do estudo

Na validade de face, os juízes avaliaram a adequabilidade do conteúdo ao público que utilizará a escala, se a estrutura e conteúdo do domínio estavam corretos e se o conteúdo era representativo^(9)^. Na validade de conteúdo, os itens da ERLAM foram avaliados conforme os critérios:

- Clareza de linguagem: o quanto a linguagem do item é suficientemente clara, compreensível e adequada para profissionais de saúde?
- Pertinência prática: qual a importância do item para o instrumento?
- Relevância teórica: qual a associação entre o item e o construto (risco de lesão de pele relacionada a adesivo médico)?

#### Instrumentos utilizados para a coleta das informações

Nas rodadas de validação foram utilizados formulários *online* que continham todos os itens da escala. Na validade de face, as opções “concordo” ou “discordo” deveriam ser assinaladas para cada item. Quando ocorria a não concordância com algum dos itens propostos na escala (ERLAM), a justificativa era relatada em espaço próprio, pelo juiz. As respostas foram comparadas para verificação da concordância entre juízes^(10)^. Na validade de conteúdo, os itens deveriam ser pontuados de um (1) a cinco (5), sendo 1 = pouquíssima, 2 = pouca, 3 = média, 4 = muita e 5= muitíssima. Itens pontuados de 1 a 3 deviam ser justificados pelos juízes no espaço “observação”.

#### Coleta de dados

Foram realizadas rodadas de validação, nas quais os instrumentos eram enviados aos juízes e retornavam aos pesquisadores. A escala era modificada conforme sugestões dos juízes e submetida a nova rodada de validação. Este processo se repetiu até atingir os índices de concordância e de coeficiente de validade de conteúdo (CVC) apropriados.

#### Tratamento e análise dos dados

Na validade de face, os itens que obtiveram índice de concordância igual ou maior a 80% foram aceitos^(11)^. Os itens menores que 80% foram ajustados conforme sugestões e enviados para os juízes para mais uma rodada de análise. Os que permaneceram menor do que o índice recomendado após os ajustes foram excluídos ou novamente readequados gerando novas rodadas de análise. Na validade de conteúdo, calculou-se o CVC para identificar os itens que não atendiam aos objetivos do instrumento^(12)^.

#### Aspectos éticos

Este estudo seguiu as diretrizes éticas e normas regulamentadoras para pesquisas com etapas em ambiente virtual. A pesquisa ocorreu somente após aprovação do Comitê de Ética em Pesquisa (nº 4.788.062 e CAAE: 44205421.3.0000.8667). Todos os juízes participantes assinaram um termo de consentimento livre e esclarecido.

### Etapa 3- Estudo observacional: avaliação de confiabilidade interobservadores e da validade de critério preditiva

#### Local da coleta de dados

Para a avaliação de confiabilidade interobservadores e da validade de critério preditiva da ERLAM, realizou-se um estudo observacional, longitudinal em três unidades de terapia intensiva (duas adultos gerais e uma coronariana) e uma unidade de clínica cirúrgica de um hospital público, de ensino, em Uberaba - MG, no Brasil.

#### Período

Antes de iniciar o estudo observacional para validade de critério preditiva e interobservadores, foi realizado pré-teste com 32 pacientes de 14/02/2023 a 09/03/2023 (não incluídos na amostra do estudo). O estudo observacional ocorreu de 09/03/2023 a 28/10/2023.

#### População

A validade de critério preditiva e confiabilidade interobservadores foi realizada com todos os pacientes admitidos nas unidades: Unidade de Terapia Intensiva Adulto (UTI-A), Unidade de Terapia Intensiva Coronariana (UTI-C), Unidade de Terapia Intensiva do tipo II (UTI-II) e Clínica Cirúrgica (CC) no período de coleta de dados, que atendessem aos critérios de inclusão.

#### Critérios de seleção

Foram incluídos todos os pacientes com 18 anos ou mais, em uso de, pelo menos, um adesivo médico fixado na pele. Foram excluídos aqueles com presença de alterações prévias da integridade da pele que comprometessem a avaliação no local de fixação dos adesivos.

#### Variáveis do estudo

Para validade de critério preditiva e interobservadores foram registrados os escores da escala ERLAM, assim como os dados referentes à caracterização do participante, aos adesivos médicos utilizados e ao surgimento das lesões.

#### Instrumentos utilizados para a coleta das informações

Para registrar as variáveis analisadas no estudo, utilizou-se um instrumento de coleta de dados elaborado pelos pesquisadores composto por características sociodemográficas do paciente (sexo, idade, setor de internação, data de admissão, diagnóstico médico, datas do primeiro e último dia de coleta de dados e motivo do encerramento da coleta de dados), adesivos médicos em uso (tipo de adesivo, local de fixação, data de inserção e data de remoção), escala ERLAM, e dados acerca das MARSI (se possui lesão, data em que a lesão foi identificada, tipo de lesão, local da lesão e adesivo médico preditor da lesão). O instrumento encontra-se disponível em https://doi.org/10.48331/SCIELODATA.RCBYDO.

#### Coleta de dados

Os critérios de identificação e classificação das MARSI ainda não são amplamente difundidos entre os profissionais de enfermagem da prática clínica^(13)^. Portanto, para evitar risco de viés no processo de identificação do surgimento de MARSI nos pacientes avaliados na pesquisa, as etapas de validade de critério preditiva e interobservadores foram realizadas por duas enfermeiras (uma mestranda e uma doutoranda), pertencentes ao Grupo de Estudo e Pesquisa em Prática Baseada em Evidências e a Segurança do Paciente no Processo do Cuidar, as quais passaram por treinamento para avaliar o surgimento de MARSI conforme os critérios preconizados por McNichol e colegas^(1)^.

O treinamento ocorreu antes do início da pesquisa e teve duração seis horas diárias por três dias, até que houvesse padronização nas avaliações da pele dos pacientes, nivelando a sua capacidade para identificar a presença de MARSI. Ressalta-se que ambas também são profissionais que atuam na prática clínica da enfermagem.

Na análise interobservadores, as pesquisadoras realizavam a avalição da superfície corporal dos pacientes nas áreas de fixação dos adesivos e aplicaram a escala (ERLAM) simultaneamente sem que houvesse comunicação entre elas. As avaliações ocorreram durante a troca ou remoção dos adesivos após o banho. Este procedimento era realizado uma vez ao dia, até a obtenção de um dos seguintes desfechos: alta do paciente do setor, transferência de leito, óbito, ausência de adesivos, completasse sete dias de acompanhamento ou houvesse surgimento de MARSI. A pele dos pacientes não foi avaliada nas trocas de adesivos que ocorreram fora dos horários de banho devido à inviabilidade da permanência das pesquisadoras no setor por período integral. Nesta etapa, o risco de viés foi minimizado, pois neste momento as pesquisadoras não tinham acesso aos pontos de corte para alto ou baixo risco de MARSI propostos pela ERLAM.

#### Tratamento e análise dos dados

Para a validade de critério preditiva, o cálculo de tamanho amostral considerou uma prevalência de lesão de 30%, precisão de 5%, intervalo de confiança de 95% e perda amostral de 20%, o que gerou uma amostra mínima de 322 participantes e máxima de 414. O cálculo do tamanho amostral, para a análise de confiabilidade interobservador, considerou um coeficiente de correlação intraclasse (ICC) esperado de 0,9, entre os escores da ERLAM, admitindo-se que este não seja inferior a 0,75%, para um poder de 80%, com nível de significância α = 0,05^(14)^. Inserindo esses valores no aplicativo *Power Analysis and Sample Size* (PASS), versão 15, obteve-se um tamanho amostral mínimo de n = 27 pacientes.

A análise da confiabilidade interobservador foi realizada por meio do coeficiente Kappa^(15)^. A análise da validade de critério preditiva foi realizada por meio de regressão logística univariada. A determinação do ponto de corte para os melhores valores de sensibilidade e especificidade da escala ocorreu por meio da análise da curva *Receiver Operating Characteristic* (ROC) (disponível em https://doi.org/10.48331/SCIELODATA.RCBYDO). Para o escore de risco total de desenvolvimento de lesão associada ao uso de adesivos médicos, utilizou-se o ICC^(14)^. Desta forma, foi possível medir a precisão da ERLAM na predição da ocorrência de MARSI.

#### Aspectos éticos

Esta pesquisa seguiu as diretrizes éticas e normas regulamentadoras para pesquisas que envolvem seres humanos e ocorreu somente após aprovação do Comitê de Ética em Pesquisa (nº 4.788.062 e CAAE: 44205421.3.0000.8667). Os pacientes que participaram das etapas de validade de critério preditiva e confiabilidade interobservador, ou seus representantes legais, assinaram o termo de consentimento livre e esclarecido.

## Resultados

A revisão integrativa resultou na inclusão de 14 artigos que abordavam fatores de risco preditores de MARSI em adultos. Foram selecionados 41 fatores de risco e agrupados em oito domínios e que compuseram a versão inicial da ERLAM (Domínio 1 - Aspectos epidemiológicos e clínicos; Domínio 2 - Características da pele em local de fixação do adesivo; Domínio 3 - Medicamentos em uso; Domínio 4 - Dispositivos médicos em uso; Domínio 5 - Tipos de adesivos em uso; Domínio 6 - Local de fixação do adesivo; Domínio 7 - Uso do adesivo; Domínio 8 - Hospitalização). Esta versão, foi encaminhada aos juízes para a realização da validação de face e conteúdo.

A primeira rodada de validação de face contou com a participação de sete juízes. Desses, quatro deixaram de participar no transcorrer das rodadas de validação, chegando ao número de três juízes na última rodada da validação de conteúdo. Após três rodadas de validação de face e modificação dos itens conforme sugestão dos juízes (Figura 1), houve concordância de 100% dos juízes quanto aos itens da escala. A redução do número de juízes não interferiu no resultado da validação, visto que o número final de juízes (três), ainda permaneceu dentro do mínimo preconizado pela literatura^(12)^.

**Figura 1-:** Sugestões de modificações dos itens da Escala de Avaliação de Risco para o Desenvolvimento de Lesões de pele Relacionadas a Adesivos Médicos nas rodadas de validação de face. Uberaba, MG, Brasil, 2022

**Item (% concordância entre juízes)**	**Sugestão de modificação**	**Versão final do item**
**Primeira rodada de validação de face (7 juízes)**		
Paciente oncológico (57,1%)	Paciente em realização de rádio e/ou quimioterapia	Paciente oncológico*
	Paciente com câncer	
Pós-operatório de cirurgia de quadril (57,1%)	Pós-operatório de cirurgia de grande porte	Pós-operatório de cirurgia de grande porte
**Segunda rodada de validação de face (5 juízes)**		
Pós-operatório de cirurgia de grande porte (60%)	Algumas cirurgias de grande porte, como a ortopédica, não utilizam adesivos para oclusão do curativo	Pós-operatório de cirurgia de grande porte*
Adesivos em local recorrente (60%)	Remoção frequente	Adesivos em local recorrente*
**Terceira rodada de validação de face (3 juízes)**		
Temperatura corporal ≥ 37.5°C (66,6%)	Em estado febril provavelmente seja mais difícil.	Temperatura corporal ≥ 37,5°C*
Sonda enteral/nasogástrica (66,6%)	Trocar o termo cateter por sonda	Sonda enteral/nasogástrica*
Sonda vesical de demora (66,6%)	Trocar o termo cateter por sonda	Sonda vesical de demora*
Fita de acrilato (66,6%)	Adicione um exemplo ao item	Fita adesiva de acrilato (ex: Medipore ^®^ , Durapore ^®^ )
Membro superior (66,6%)	Subdividir o membro superior em partes, visto que algumas áreas são mais vulneráveis à lesão que outras	Membro superior*

*Após contato com os juízes foi consentida a manutenção do termo

Na primeira rodada da validação de conteúdo, obteve-se CVC total de 0,94 e CVC final de cada item ≥ 0,88 para todos os aspectos avaliados (clareza, relevância teórica e pertinência prática), o que corresponde à aprovação na íntegra dos itens. Portanto, para 94% dos avaliadores, a ERLAM foi considerada relevante para a predição do risco de desenvolvimento de MARSI em adultos hospitalizados.

Assim, obteve-se a versão final da ERLAM, composta por 48 itens e oito domínios (Figura 2).

Para obtenção do escore total da escala deve ser somado um ponto para cada item identificado no paciente, dessa forma, o escore da ERLAM pode variar de zero a 48 pontos. Para classificação do risco de MARSI em alto e baixo, o escore adotado pela curva ROC foi de 11 pontos, visto que este valor apresentou melhor relação entre a sensibilidade e especificidade da escala. Desta forma, os escores < 11 classificam os pacientes em baixo risco e os escores em alto risco de desenvolver MARSI.

Para análise da validade de critério preditiva da ERLAM foram avaliados 329 pacientes e para a confiabilidade interobservadores, 32. Dentre os participantes, 193 (58,7%) eram do sexo masculino, a idade mínima foi de 19 anos e a máxima de 91 anos, com média de 57. Pacientes com 60 anos ou mais representaram 54,5% da amostra. Cento e dez (33,4%) estavam internados na clínica cirúrgica, 110 (33,4%) na UTI- C ou UTI - II e 109 (33,2%) na UTI-A.

Os escores obtidos da ERLAM, durante o estudo observacional da etapa de validade de critério preditiva, variaram de quatro a 27 pontos. Ao todo, 143 (43,5%) pacientes desenvolveram algum tipo de MARSI. A regressão logística da associação dos escores da ERLAM com a ocorrência de MARSI constatou que a média do escore foi de 15,07 pontos entre os pacientes que desenvolveram lesão e de 10,90 pontos entre os 186 (56,5%) pacientes que não desenvolveram lesão (Tabela 1).

**Figura 2-:** Versão final da Escala de Avaliação de Risco para o Desenvolvimento de Lesões de pele Relacionadas a Adesivos Médicos

**Tabela 1-:** Regressão logística da associação dos escores da Escala de Avaliação de Risco para o Desenvolvimento de Lesões de pele Relacionadas a Adesivos Médicos com a ocorrência de lesões de pele relacionadas a adesivos médicos. Uberaba, MG, Brasil, 2023

Ocorrência de MARSI*	**n** ^†^ **(%)**	**Min.-máx.**	**Média**	**Desvio-Padrão**	**Mediana**	**RC** ^‡^	**IC** ^§^ **(95%)**
Sim	143 (43,5)	4 - 24	15,07	4,11	15,0		
						1,27	1,19-1,35
Não	186 (56,5)	4 - 27	10,90	4,10	10,0		

*MARSI = Lesões de pele relacionadas a adesivos médicos; ^†^n = Número de participantes; ^‡^RC = Razão de chances; ^§^IC = Intervalo de confiança

A ERLAM apresentou uma razão de chances (RC) de 1,27, o que demonstra associação estatisticamente significativa (p < 0,001) entre o escore gerado pela escala e a ocorrência de MARSI. Para cada ponto registrado no escore, há um aumento de 27,0% de chance de ocorrência de MARSI. Portanto, a ERLAM possui validade de critério preditiva associada à ocorrência de MARSI em adultos hospitalizados. Além disso, as análises também demonstraram que pacientes classificados como alto risco para MARSI, segundo a classificação da ERLAM, têm 8,14 vezes mais chances de desenvolverem MARSI durante a hospitalização do que aqueles classificados como baixo risco (Tabela 1). A regressão logística entre a classificação de risco da ERLAM, com ponto de corte o escore de 11 pontos, e a ocorrência de MARSI apresentou sensibilidade de 86% e especificidade de 57% (Tabela 2).

**Tabela 2-:** Relação da classificação de risco da Escala de Avaliação de Risco para o Desenvolvimento de Lesões de pele Relacionadas a Adesivos Médicos e a ocorrência de lesões de pele relacionadas a adesivos médicos. Uberaba, MG, Brasil, 2023

	ERLAM*		
Presença de MARSI ^†^	Alto risco para MARSI ^†^ n ^‡^ (%)	Baixo risco para MARSI ^†^ n ^‡^ (%)	Total n ^‡^ (%)
Sim	123 (86,00%)	20 (14,00%)	143 (100,00%)
Não	80 (43,00%)	106 (57,00%)	186 (100,00%)
Total n (%)	203 (61,70%)	126 (38,30%)	329 (100,00%)

*ERLAM = Escala de Avaliação de Risco para o Desenvolvimento de Lesões de pele Relacionadas a Adesivos Médicos; ^†^MARSI = Lesões de pele relacionadas a adesivos médicos; ^‡^n = Número de participantes

A confiabilidade interobservadores demonstrou que 39 (81,5%) itens da escala apresentaram valores de Kappa correspondentes aos melhores índices de concordância, sendo 30 (62,5%) itens com concordância perfeita e nove (19,0%) com concordância substancial. Todos os itens da ERLAM atingiram níveis de significância p < 0,05, sendo a maioria p = 0,001. O nível de concordância entre os observadores variou de 75% a 100,0%. A análise de confiabilidade interobservador da ERLAM (Tabela 3) obteve um ICC de 0,92, classificado como excelente^(13)^ e um p < 0,05, o que demonstra a alta confiabilidade interobservador da escala.

**Tabela 3-:** Análise de confiabilidade interobservadores da Escala de Avaliação de Risco para o Desenvolvimento de Lesões de pele Relacionadas a Adesivos Médicos. Uberaba, MG, Brasil, 2023

**Observador**	**Mín.**	**Máx.**	**Média**	**Mediana**	**Desvio-Padrão**	ICC*	***p*** ^†^
**Obs. 1**	9	22	14,38	14,0	3,36	0,92	< 0,05
**Obs. 2**	9	22	13,81	14,0	3,22		

*ICC = Coeficiente de correlação intraclasse; ^†^ *p* = Nível de significância

## Discussão

Após busca na literatura, não foi identificada a existência de uma escala que classifique o risco de desenvolvimento de MARSI em adultos hospitalizados. Quanto à validade de conteúdo, a ERLAM apresentou valores significativos, aproximados aos das escalas que avaliam risco de lesões de pele, como a Escala de Avaliação de Risco para o Desenvolvimento de Lesões Decorrentes do Posicionamento Cirúrgico do Paciente (ELPO)^(16)^, e da Escala de avaliação de risco de lesão de pele relacionada ao adesivo no local de inserção do PICC em pacientes oncológicos^(17)^. Nessas escalas o índice de validade de conteúdo (IVC) variou de 0,83^(17)^ a 0,88^(16)^.

Na ERLAM, a diferença entre os escores dos grupos classificados como alto ou baixo risco para MARSI foi de mais de quatro pontos no escore total, próximo ao apresentado pela ELPO, que obteve diferença entre grupos com ou sem lesão por pressão (LP) de quase cinco pontos (p < 0,001)^(16)^. As análises das propriedades métricas da ERLAM também apresentaram excelentes resultados de acurácia, sensibilidade, especificidade e RC. Valores que também se aproximaram aos apresentados no processo de validação das demais escalas. A ELPO^(16)^ apresentou acurácia de 0,83, sensibilidade de 88,0%, especificidade de 64,4% e RC de 1,44. Já as propriedades métricas da Braden^(18)^ sofreram variações de acordo com o nível de escolaridade dos profissionais que utilizaram a escala. Portanto, apesar da sensibilidade da Braden ser de 100%, a especificidade variou de 64% a 90% entre técnicos e enfermeiros, respectivamente. A escala de avaliação de risco de lesão de pele relacionada ao adesivo no local de inserção do PICC em pacientes oncológicos^(17)^ apresentou acurácia de 0,75, sensibilidade 80,0% e especificidade de 65,5%. Já a escala de avaliação de risco para MARSI em bebês e crianças de unidade de terapia intensiva^(19)^ apresentou uma acurácia de 0,92, sensibilidade de 86,27% e especificidade de 98,76%.

Ressalta-se que os valores de sensibilidade e especificidade de um instrumento podem sofrer variações após validado, de acordo com a população em que é aplicado. A exemplo da Escala Braden, que ao ser aplicada em diferentes estudos após validada, apresentou valores de sensibilidade que variaram de 31,2% a 95% e de especificidade com variação de 16,6% a 88,2%^(20)^.

A confiabilidade interobservador da ERLAM atingiu excelentes resultados assim como dos demais instrumentos, adotado o valor referência de ICC ≥ 0,75^(14)^. A confiabilidade de Braden variou de acordo com o grau de instrução do profissional que utilizou a escala (0,83 a 0,94 para auxiliares de enfermagem e 0,99 para enfermeiros)^(18)^. A ELPO apresentou ICC de 0,99^(16)^, enquanto a escala de avaliação de risco de lesão de pele relacionada ao adesivo no local de inserção do PICC em pacientes oncológicos^(17)^ teve um ICC de 0,97 e a escala de avaliação de risco para MARSI em bebês e crianças de unidade de terapia intensiva^(19)^ um ICC de 0,85.

Identificou-se como limitação do estudo a ausência de outra escala de avaliação de risco para MARSI em adultos a fim de realizar a validade concorrente. A ERLAM foi criada e validada no idioma português do Brasil. Portanto, é necessário que novos estudos sejam realizados para traduzir e validar o uso da escala em outros idiomas e, assim, possa ser utilizada em outros países.

A ERLAM poderá auxiliar na prática de enfermagem baseada em evidências, pois reuniu de forma objetiva as melhores evidências científicas acerca dos fatores de risco para MARSI em adultos hospitalizados. Além disso, ao classificar essa população quanto ao risco de desenvolvimento dessas lesões, a escala permitirá que os enfermeiros identifiquem pacientes em risco para a ocorrência de MARSI com maior rapidez e precisão.

## Conclusão

Por meio das análises de validade de critério preditiva e confiabilidade interobservadores, a ERLAM demonstrou excelente acurácia, sensibilidade e especificidade. Logo, este é um instrumento válido e confiável para identificar o risco da ocorrência de MARSI em adultos hospitalizados.

## Declaração de Disponibilidade de Dados

Todos os dados gerados ou analisados durante este estudo estão incluídos neste artigo publicado.

# Translation: Escala de Evaluación del Riesgo de Desarrollo de Lesiones Cutáneas Relacionadas con Adhesivos Médicos*

**DOI:** 10.1590/1518-8345.7891.4709

**Authors:** Maíla Fidalgo de Faria, Talita Silva Alves Tibola, Maria Beatriz Guimarães Raponi, Patrícia da Silva Pires, Maria Sagrario Gómez Cantarino, Maria Helena Barbosa

**Affiliations:** 1 Universidade Federal do Triângulo Mineiro, Uberaba, MG, Brasil.; 2 Becaria de la Coordenação de Aperfeiçoamento de Pessoal de Nível Superior (CAPES), Brasil.; 3 Universidade Federal de Uberlândia, Faculdade de Medicina, Escola de Enfermagem, Uberlândia, MG, Brasil.; 4 Universidade Federal da Bahia, Escola de Enfermagem, Vitória da Conquista, BA, Brasil.; 5 Universidade de Castilla-La Mancha, Escola de Fisioterapia e Enfermagem, Toledo, Castilla-La Mancha, España.; 6 Becaria del Conselho Nacional de Desenvolvimento Científico e Tecnológico (CNPq), Brasil.

**Keywords:** Adhesivos, Heridas y Lesiones, Piel, Seguridad del Paciente, Enfermería Basada en la Evidencia, Prevención Secundaria

## Abstract

desarrollar y validar la Escala de Evaluación del Riesgo para el Desarrollo de Lesiones Cutáneas Relacionadas con Adhesivos Médicos en adultos hospitalizados.

estudio metodológico que comprendió la elaboración, validación de apariencia, de contenido, de criterio predictivo y análisis de confiabilidad interobservadores de la escala. Fue elaborada utilizando factores de riesgo obtenidos en una revisión integrativa y pasó por validación de apariencia y contenido por siete jueces. Trescientos veintinueve pacientes participaron en la validez de criterio predictivo y 32 en la confiabilidad.

la escala consta de 48 ítems y ocho dominios. Los puntajes < 11 clasifican a los pacientes en bajo riesgo y los ≥ 11 en alto riesgo de desarrollar lesiones por adhesivos médicos. En la validez de apariencia hubo concordancia del 100 % de los jueces; en la de contenido se obtuvo un coeficiente de validez de contenido total de 0,94. En la validez de criterio predictivo hubo asociación estadísticamente significativa (p < 0,001) entre el puntaje generado por la escala y la ocurrencia de lesiones por adhesivos médicos. El análisis de confiabilidad mostró un coeficiente de correlación intraclase de 0,92.

la escala desarrollada es un instrumento válido y confiable para identificar el riesgo de ocurrencia de lesiones por adhesivos médicos en adultos hospitalizados.

## Destacados

**(1)** La ERLAM es un instrumento que posee excelente exactitud, sensibilidad y especificidad. **(2)** La ERLAM es un instrumento válido y confiable para identificar el riesgo de MARSI. **(3)** La ERLAM ayudará en la prevención de MARSI en adultos hospitalizados. **(4)** La ERLAM está validada y lista para su uso en portugués de Brasil.

## Introducción

Las lesiones de piel relacionadas con adhesivos médicos (*medical adhesive-related skin injury* - MARSI) se caracterizan por anomalías presentes en la piel después de 30 minutos o más de la retirada del adhesivo^(1)^. Estos son eventos adversos evitables que están presentes en cualquier escenario asistencial y comprometen el confort y la seguridad del paciente, así como la calidad de la atención de enfermería. Además, estas lesiones son actos iatrogénicos que pueden desencadenar implicaciones éticas y legales para los profesionales y las instituciones de salud^(2)^.

Las MARSI poseen diversas etiologías y se clasifican en lesiones mecánicas (desnudamiento, ampollas y lesiones por fricción), dermatitis (de contacto irritante y alérgica) y otras causas (maceración y foliculitis)^(1)^. La incidencia de estas lesiones varía según la población evaluada, con registros en la literatura que oscilan entre 0,1% y 60,3%^(3)^.

La identificación de los factores de riesgo es fundamental para la prevención de las MARSI. Sin embargo, debido a la ausencia de instrumentos validados para predecir la ocurrencia de estas lesiones, algunos estudios utilizaron la Escala de Braden para intentar predecir MARSI^(2)^. No obstante, existen limitaciones, ya que no se trata de un instrumento específico para esta evaluación. Cabe destacar que las clasificaciones de riesgo deben realizarse mediante instrumentos validados que garanticen niveles adecuados de especificidad y sensibilidad. La actualización del consenso de especialistas sobre MARSI incluye la evaluación del paciente respecto al riesgo de desarrollar MARSI como uno de los principales pilares para la prevención de estas lesiones^(4)^. Por ello, es necesario elaborar y validar un instrumento capaz de clasificar a los pacientes según el riesgo de desarrollar dichas lesiones durante la hospitalización.

Los factores intrínsecos o extrínsecos pueden aumentar la predisposición a la aparición de MARSI. Entre los factores de riesgo intrínsecos se destacan: edad > 50 años; bajo nivel de albúmina sérica; edema cutáneo en el lugar de fijación del adhesivo; antecedente de MARSI; piel seca; antecedentes de alergias cutáneas; elevada humedad de la piel; Diabetes mellitus; bajo peso corporal; hipoproteinemia; presencia de comorbilidades; problemas cutáneos; antecedentes de alergias alimentarias; elasticidad cutánea disminuida; fiebre. Entre los factores de riesgo extrínsecos figuran: antecedente de tabaquismo; uso de sedantes; alta frecuencia de uso de adhesivos; tipo de adhesivo utilizado; uso de inmunosupresores; uso de anticoagulantes; ventilación mecánica; puntuación elevada en la escala de Braden^(5)^.

Considerando la necesidad de identificar factores de riesgo para implementar medidas que prevengan las MARSI, este estudio tuvo como objetivo general desarrollar y validar la Escala de Evaluación del Riesgo para el Desarrollo de Lesiones Cutáneas Relacionadas con Adhesivos Médicos en adultos hospitalizados. Los objetivos específicos fueron: realizar una revisión integrativa para reunir evidencias científicas que respaldaran el desarrollo de la escala; llevar a cabo la validación aparente y de contenido de la escala elaborada; evaluar la validez de criterio predictivo de la escala; y analizar la confiabilidad interobservadores de la escala desarrollada.

## Método

### Diseño del estudio

Se trata de un estudio metodológico de construcción y validación de una escala, conducido en tres etapas:

- Etapa 1- Estudio de revisión: revisión integrativa de los factores de riesgo para MARSI en adultos hospitalizados;
- Etapa 2- Estudio metodológico: desarrollo de la ERLAM a partir de los resultados de la revisión integrativa y validación de apariencia y contenido de la escala elaborada;
- Etapa 3- Estudio observacional: análisis de propiedades métricas (validez de criterio predictiva y confiabilidad interobservadores) de la versión final de la ERLAM.

### Etapa 1 - Estudio de revisión (revisión integrativa)

#### Lugar de recolección de datos

Para la construcción de los ítems de la escala se utilizaron los factores de riesgo obtenidos mediante una revisión integrativa registrada en *Open Science Framework*, DOI: https://doi.org/10.17605/OSF.IO/346YF. La revisión se realizó en las bases de datos *PubMed*, *Cochrane Library*, *Embase*, LILACS, CINAHL y en el buscador *Google Scholar*.

#### Período

Las búsquedas de los artículos se realizaron en octubre de 2021.

#### Criterios de selección

La revisión integrativa se condujo conforme a las etapas recomendadas en la literatura^(6)^ y en la *guideline* PRISMA^(7)^. Para atender al objetivo de la revisión, el mnemónico PICo se estructuró de la siguiente forma:

- Población (P) = adultos hospitalizados
- Interés (I) = factores de riesgo para la ocurrencia de MARSI
- Contexto (Co) = utilización de adhesivos médicos en la atención de salud

A partir del mnemónico elaborado, se estableció la siguiente pregunta de revisión: ¿cuáles son los factores de riesgo para la ocurrencia de MARSI en adultos hospitalizados que utilizan adhesivos médicos durante la atención de salud? Se adoptaron como criterios de inclusión estudios primarios que respondieran a la pregunta de revisión, en inglés, portugués o español, sin delimitación de año de publicación. Se excluyeron artículos que abordaban exclusivamente a niños, resúmenes de eventos, capítulos de libros, cartas, manuales, editoriales, métodos de revisión, relatos de caso, artículos duplicados y aquellos que no contemplaban el tema en estudio. El diagrama PRISMA fue depositado en https://doi.org/10.48331/SCIELODATA.RCBYDO.

#### Instrumentos utilizados para la recolección de la información

Para la extracción de datos y síntesis de evidencias se elaboró una tabla que contenía: autor/autores; año de publicación; objetivos; población y tamaño muestral; método; tipo y duración de la intervención (si la hubiera); principales hallazgos relacionados con los factores de riesgo para MARSI en adultos hospitalizados; nivel de evidencia. La clasificación del nivel de evidencia siguió los criterios del *Oxford Centre for Evidence-Based Medicine*, que utiliza una clasificación de 1 a 5, siendo 1 el mayor nivel de evidencia y 5 el menor^(8)^.

#### Recolección de datos

Los artículos identificados en la búsqueda se exportaron a la aplicación gratuita *Rayyan* QCRI y fueron seleccionados mediante el análisis del título y el resumen, individualmente por dos investigadoras, de forma ciega, conforme a los criterios de inclusión y exclusión adoptados.

Tras esta selección, se realizó una reunión de consenso entre las investigadoras y un tercer evaluador, en la cual se obtuvo la lista de artículos que fueron leídos en su totalidad. Las listas de referencias de los artículos seleccionados también se consultaron y aquellos que cumplían los criterios de inclusión se añadieron a la muestra final de artículos.

#### Tratamiento y análisis de los datos

Los factores de riesgo identificados en la revisión integrativa se agruparon en categorías. Se consideraron factores de riesgo las variables presentadas en los estudios como predictores significativos para la ocurrencia de MARSI (p < 0,05), los factores comunes presentados en más de un estudio, así como los criterios establecidos en el consenso elaborado por McNichol y colaboradores^(1)^ para la evaluación, prevención y tratamiento de MARSI.

#### Aspectos éticos

Este estudio cumplió todos los preceptos éticos relativos a los derechos de autor de los artículos incluidos en la etapa de revisión.

### Etapa 2 - Estudio metodológico: validación de apariencia y contenido

#### Lugar de recolección de datos

Las etapas de validación de apariencia y contenido de la ERLAM se llevaron a cabo en línea mediante formularios enviados a los correos electrónicos de los jueces seleccionados.

#### Período

Tras ser elaborada, la ERLAM pasó por rondas de validación de apariencia y contenido, en el período del 21/06/2022 al 19/12/2022.

#### Población

Fueron invitados a participar como jueces en el proceso de validación de la escala los profesionales que cumplieran los siguientes criterios de inclusión: ser enfermeros, doctores, con actuación en el área hospitalaria y producción científica relacionada con lesiones cutáneas.

#### Criterios de selección

La búsqueda de los profesionales que cumplían los criterios de inclusión se realizó en la Plataforma Lattes, utilizando la palabra clave “*Pele*” en la búsqueda por asunto. Se empleó el filtro “*atuação profissional*” con gran área “*Ciências da Saúde*”, área “*enfermagem*” y subáreas “*enfermagem em saúde do adulto e idoso*”. También se hizo una segunda búsqueda con la palabra clave “*feridas*”. Se enviaron correos electrónicos con invitación para participar en la validación de la escala a los 37 profesionales identificados en la búsqueda que cumplían los criterios de inclusión. Sin embargo, tras tres intentos de contacto, solo siete aceptaron participar en las etapas de validación de apariencia y contenido de la ERLAM.

#### Variables del estudio

En la validez de apariencia, los jueces evaluaron la adecuación del contenido al público que utilizará la escala, si la estructura y contenido del dominio estaban correctos y si el contenido era representativo^(9)^. En la validez de contenido, los ítems de la ERLAM fueron evaluados conforme a los criterios:

- Claridad del lenguaje: ¿qué tan clara, comprensible y adecuada es la redacción del ítem para los profesionales de salud?
- Pertinencia práctica: ¿cuál es la importancia del ítem para el instrumento?
- Relevancia teórica: ¿cuál es la relación del ítem con el constructo (riesgo de lesión cutánea relacionada con adhesivos médicos)?

#### Instrumentos utilizados para la recolección de la información

En las rondas de validación se utilizaron formularios en línea que contenían todos los ítems de la escala. En la validez de apariencia, las opciones “de acuerdo” o “en desacuerdo” debían ser marcadas para cada ítem. Cuando se señalaba desacuerdo con alguno de los ítems propuestos en la escala (ERLAM), el juez debía justificarlo en un espacio propio. Las respuestas se compararon para verificar la concordancia entre jueces^(10)^. En la validez de contenido, los ítems debían ser puntuados de 1 a 5, siendo 1 = poquísima, 2 = poca, 3 = media, 4 = mucha y 5 = muchísima. Los ítems puntuados de 1 a 3 debían ser justificados por los jueces en el espacio “observación”.

#### Recolección de datos

Se realizaron rondas de validación en las que los instrumentos se enviaban a los jueces y regresaban a los investigadores. La escala se modificaba según las sugerencias de los jueces y se sometía a una nueva ronda de validación. Este proceso se repitió hasta alcanzar los índices de concordancia y el coeficiente de validez de contenido (CVC) apropiados.

#### Tratamiento y análisis de los datos

En la validez de apariencia, los ítems que obtuvieron un índice de concordancia igual o mayor al 80% fueron aceptados^(11)^. Los ítems con menos del 80% fueron ajustados conforme a las sugerencias y enviados a los jueces para otra ronda de análisis. Los que permanecieron por debajo del índice recomendado tras los ajustes fueron excluidos o nuevamente readecuados, generando nuevas rondas de análisis. En la validez de contenido, se calculó el CVC para identificar los ítems que no cumplían los objetivos del instrumento^(12)^.

#### Aspectos éticos

Este estudio siguió las directrices éticas y las normas reglamentarias para investigaciones con etapas en entorno virtual. La investigación se llevó a cabo solo después de la aprobación del Comité de Ética en Investigación (n.º 4.788.062 y CAAE: 44205421.3.0000.8667). Todos los jueces participantes firmaron un término de consentimiento informado.

### Etapa 3 – Estudio observacional: evaluación de la confiabilidad interobservadores y de la validez de criterio predictiva

#### Lugar de recolección de datos

Para la evaluación de la confiabilidad interobservadores y de la validez de criterio predictiva de la ERLAM, se llevó a cabo un estudio observacional, longitudinal, en tres unidades de cuidados intensivos (dos de adultos generales y una coronaria) y una unidad de clínica quirúrgica de un hospital público y universitario de Uberaba, MG, Brasil.

#### Período

Antes de iniciar el estudio observacional para la validez de criterio predictivo e interobservadores, se realizó una prueba piloto con 32 pacientes, del 14/02/2023 al 09/03/2023 (no incluidos en la muestra del estudio). El estudio observacional se desarrolló del 09/03/2023 al 28/10/2023.

#### Población

La validez de criterio predictiva y la confiabilidad interobservadores se realizaron con todos los pacientes admitidos en las unidades: Unidad de Cuidados Intensivos para Adultos (UCI-A), Unidad de Cuidados Intensivos Coronarios (UCI-C), Unidad de Cuidados Intensivos Tipo II (UCI-II) y Clínica Quirúrgica (CC) durante el período de recolección de datos que cumplieron los criterios de inclusión.

#### Criterios de selección

Se incluyó a todos los pacientes de 18 años o más que utilizaban al menos un adhesivo médico fijado a la piel. Se excluyeron aquellos con alteraciones previas de la integridad cutánea que comprometieran la evaluación en el sitio de fijación de los adhesivos.

#### Variables del estudio

Para la validez de criterio predictivo e interobservadores se registraron los puntajes de la escala ERLAM, así como los datos referentes a la caracterización del participante, a los adhesivos médicos utilizados y a la aparición de las lesiones.

#### Instrumentos utilizados para la recolección de la información

Para registrar las variables analizadas se utilizó un formulario de recolección de datos elaborado por los investigadores que incluyó características sociodemográficas del paciente (sexo, edad, unidad de internación, fecha de admisión, diagnóstico médico, fechas del primer y último día de recolección y motivo de finalización de la recolección), adhesivos médicos en uso (tipo de adhesivo, sitio de fijación, fecha de inserción y fecha de retirada), la ERLAM y datos sobre las MARSI (si existe lesión, fecha de identificación, tipo de lesión, ubicación y adhesivo médico predictor de la lesión). El formulario fue depositado en https://doi.org/10.48331/SCIELODATA.RCBYDO.

#### Recolección de datos

Los criterios de identificación y clasificación de las MARSI aún no están ampliamente difundidos entre los profesionales de enfermería de la práctica clínica^(13)^. Por lo tanto, para evitar riesgo de sesgo en el proceso de identificación de la aparición de MARSI en los pacientes evaluados, las etapas de validez de criterio predictiva e interobservadores fueron realizadas por dos enfermeras (una maestranda y una doctoranda), pertenecientes al Grupo de Estudio e Investigación en Práctica Basada en Evidencias y Seguridad del Paciente en el Proceso de Cuidar, quienes recibieron entrenamiento para evaluar la aparición de MARSI conforme a los criterios preconizados por McNichol y colegas^(1)^.

La capacitación se llevó a cabo antes del inicio del estudio y tuvo una duración de seis horas diarias durante tres días, hasta lograr la estandarización en las evaluaciones cutáneas de los pacientes, nivelando su capacidad para identificar la presencia de MARSI. Cabe señalar que ambas también son profesionales que actúan en la práctica clínica de enfermería.

En el análisis interobservadores, las investigadoras evaluaban la superficie corporal de los pacientes en las áreas de fijación de los adhesivos y aplicaban la escala (ERLAM) simultáneamente, sin comunicación entre ellas. Las evaluaciones se realizaban durante el cambio o la retirada de los adhesivos después del baño. Este procedimiento se efectuaba una vez al día hasta la obtención de uno de los siguientes desenlaces: alta del paciente, traslado de cama, óbito, ausencia de adhesivos, finalización de siete días de seguimiento o aparición de MARSI. La piel de los pacientes no fue evaluada durante los cambios de adhesivos fuera del horario de baño, debido a la inviabilidad de la permanencia de las investigadoras en el sector de forma continua. En esta etapa, el riesgo de sesgo se minimizó, ya que las investigadoras no tenían acceso a los puntos de corte para alto o bajo riesgo de MARSI propuestos por la ERLAM.

#### Tratamiento y análisis de los datos

Para la validez de criterio predictivo, el cálculo del tamaño muestral consideró una prevalencia de lesión del 30%, una precisión del 5%, un intervalo de confianza del 95% y una pérdida muestral del 20%, lo que generó un tamaño mínimo de 322 participantes y máximo de 414. El cálculo del tamaño muestral para el análisis de confiabilidad interobservadores contempló un coeficiente de correlación intraclase (ICC) esperado de 0,9 entre las puntuaciones de la ERLAM, admitiéndose que este no fuera inferior al 0,75%, con un poder del 80% y un nivel de significancia α = 0,05^(14)^. Al introducir estos valores en la aplicación *Power Analysis and Sample Size* (PASS), versión 15, se obtuvo un tamaño muestral mínimo de n = 27 pacientes.

El análisis de la confiabilidad interobservadores se realizó mediante el coeficiente Kappa^(15)^. La validez de criterio predictivo se evaluó mediante regresión logística univariada. La determinación del punto de corte para los mejores valores de sensibilidad y especificidad de la escala se obtuvo mediante el análisis de la curva *Receiver Operating Characteristic* (ROC) (disponible en https://doi.org/10.48331/SCIELODATA.RCBYDO). Para la puntuación total de riesgo de desarrollo de lesiones asociadas al uso de adhesivos médicos se empleó el ICC^(14)^. Así fue posible medir la precisión de la ERLAM en la predicción de la aparición de MARSI.

#### Aspectos éticos

Esta investigación siguió las directrices éticas y normas reglamentarias para estudios que involucran seres humanos y se llevó a cabo únicamente después de la aprobación del Comité de Ética en Investigación (n.º 4.788.062 y CAAE: 44205421.3.0000.8667). Los pacientes que participaron en las etapas de validez de criterio predictivo y confiabilidad interobservadores, o sus representantes legales, firmaron el término de consentimiento informado.

## Resultados

La revisión integrativa resultó en la inclusión de 14 artículos que abordaban factores de riesgo predictores de MARSI en adultos. Se seleccionaron 41 factores de riesgo y se agruparon en ocho dominios que compusieron la versión inicial de la ERLAM (Dominio 1 – Aspectos epidemiológicos y clínicos; Dominio 2 – Características de la piel en el sitio de fijación del adhesivo; Dominio 3 – Medicamentos en uso; Dominio 4 – Dispositivos médicos en uso; Dominio 5 – Tipos de adhesivos en uso; Dominio 6 – Sitio de fijación del adhesivo; Dominio 7 – Uso del adhesivo; Dominio 8 – Hospitalización). Esta versión se envió a los jueces para la validación de apariencia y contenido.

La primera ronda de validación de apariencia contó con la participación de siete jueces. De ellos, cuatro se retiraron durante el proceso, quedando tres jueces en la última ronda de validación de contenido. Tras tres rondas de validación de apariencia y la modificación de los ítems según las sugerencias de los jueces (Figura 1), hubo concordancia del 100% de los jueces respecto a los ítems de la escala. La reducción del número de jueces no influyó en el resultado, ya que el número final (tres) se mantuvo dentro del mínimo recomendado por la literatura^(12)^.

**Figura 1-:** Sugerencias de modificaciones de los ítems de la Escala de Evaluación del Riesgo para el Desarrollo de Lesiones Cutáneas Relacionadas con Adhesivos Médicos en las rondas de validación de apariencia. Uberaba, MG, Brasil, 2022

Ítem (% de concordancia entre jueces)	**Sugerencia de modificación**	**Versión final del ítem**
**Primera ronda de validación de apariencia (7 jueces)**		
Paciente oncológico (57,1%)	Paciente en tratamiento con radio y/o quimioterapia	Paciente oncológico*
	Paciente con cáncer	
Postoperatorio de cirugía de cadera (57,1%)	Postoperatorio de cirugía de gran porte	Postoperatorio de cirugía de gran porte
**Segunda ronda de validación de apariencia (5 jueces)**		
Posoperatorio de cirugía de gran porte (60%)	Algunas cirugías de gran porte, como la ortopédica, no utilizan adhesivos para la oclusión del apósito	Posoperatorio de cirugía de gran porte*
Adhesivos en lugar recurrente (60%)	Retirada frecuente	Adhesivos en lugar recurrente*
**Tercera ronda de validación de apariencia (3 jueces)**		
Temperatura corporal ≥ 37,5°C (66,6%)	En estado febril probablemente sea más difícil.	Temperatura corporal ≥ 37,5°C*
Sonda enteral/nasogástrica (66,6%)	Cambiar el término catéter por sonda	Sonda enteral/nasogástrica*
Sonda vesical de demora (66,6%)	Cambiar el término catéter por sonda	Sonda vesical permanente*
Cinta de acrilato (66,6%)	Añadir un ejemplo al ítem	Cinta adhesiva de acrilato (p. ej., Medipore ^®^ , Durapore ^®^ )
Miembro superior (66,6%)	Subdividir el miembro superior en partes, dado que algunas áreas son más vulnerables a lesión que otras	Miembro superior*

*Tras los contactos con los jueces, se consensuó mantener el término

En la primera ronda de validación de contenido se obtuvo un CVC total de 0,94 y un CVC final de cada ítem ≥ 0,88 para todos los aspectos evaluados (claridad, relevancia teórica y pertinencia práctica), lo que corresponde a la aprobación íntegra de los ítems. Por lo tanto, para el 94% de los evaluadores, la ERLAM fue considerada relevante para la predicción del riesgo de desarrollo de MARSI en adultos hospitalizados.

Así, se obtuvo la versión final de la ERLAM, compuesta por 48 ítems y ocho dominios (Figura 2).

Para obtener la puntuación total de la escala debe sumarse un punto por cada ítem identificado en el paciente; de este modo, la puntuación de la ERLAM puede variar de cero a 48 puntos. Para clasificar el riesgo de MARSI en alto y bajo, la puntuación adoptada por la curva ROC fue de 11 puntos, dado que ese valor presentó la mejor relación entre la sensibilidad y la especificidad de la escala. De esta forma, las puntuaciones < 11 clasifican a los pacientes en bajo riesgo y las puntuaciones ≥ 11 indican alto riesgo de desarrollar MARSI.

**Figura 2-:** Versión final de la Escala de Evaluación del Riesgo para el Desarrollo de Lesiones Cutáneas Relacionadas con Adhesivos Médicos

Para el análisis de la validez de criterio predictiva de la ERLAM se evaluaron 329 pacientes y, para la confiabilidad interobservadores, 32. De los participantes, 193 (58,7%) eran de sexo masculino; la edad mínima fue de 19 años y la máxima de 91, con una media de 57,00. Los pacientes de 60 años o más representaron el 54,5% de la muestra. Ciento diez (33,4%) estaban hospitalizados en la clínica quirúrgica, 110 (33,4%) en la UCI-C o UCI-II y 109 (33,2%) en la UCI-A.

Los puntajes de la ERLAM obtenidos durante el estudio observacional de la etapa de validez de criterio predictiva variaron de cuatro a 27 puntos. En total, 143 (43,5%) pacientes desarrollaron algún tipo de MARSI. La regresión logística de la asociación de los puntajes de la ERLAM con la ocurrencia de MARSI constató que la media del puntaje fue de 15,07 puntos entre los pacientes que desarrollaron lesión y de 10,9 puntos entre los 186 (56,5%) que no la desarrollaron (Tabla 1).

**Tabla 1–:** Regresión logística de la asociación de los puntajes de la Escala de Evaluación del Riesgo para el Desarrollo de Lesiones Cutáneas Relacionadas con Adhesivos Médicos con la ocurrencia de lesiones cutáneas relacionadas con adhesivos médicos. Uberaba, MG, Brasil, 2023

Ocurrencia de MARSI*	**n** ^†^ **(%)**	**Mín.-máx.**	**Media**	**Desviación estándar**	**Mediana**	**RP** ^‡^	**IC** ^§^ **(95%)**
Sí	143 (43,5)	4 - 24	15,07	4,11	15,00		
						1,27	1,19 -1,35
No	186 (56,5)	4 - 27	10,90	4,10	10,00		

*MARSI = Lesiones de piel relacionadas con adhesivos médicos; ^†^n = Número de participantes; ^‡^RP = Razón de probabilidades; ^§^IC = Intervalo de confianza

La ERLAM presentó una razón de probabilidades (RC) de 1,27, lo que demuestra una asociación estadísticamente significativa (p < 0,001) entre la puntuación generada por la escala y la ocurrencia de MARSI. Por cada punto registrado en la puntuación, hay un aumento del 27,0% en la probabilidad de ocurrencia de MARSI. Por lo tanto, la ERLAM posee validez de criterio predictiva asociada a la ocurrencia de MARSI en adultos hospitalizados. Además, los análisis también mostraron que los pacientes clasificados como de alto riesgo de MARSI, según la clasificación de la ERLAM, tienen 8,14 veces más probabilidades de desarrollar MARSI durante la hospitalización que aquellos clasificados como de bajo riesgo (Tabla 1). La regresión logística entre la clasificación de riesgo de la ERLAM, con punto de corte en la puntuación de 11 puntos, y la ocurrencia de MARSI presentó una sensibilidad del 86% y una especificidad del 57% (Tabla 2).

**Tabla 2-:** Relación entre la clasificación de riesgo de la Escala de Evaluación del Riesgo para el Desarrollo de Lesiones Cutáneas Relacionadas con Adhesivos Médicos y la ocurrencia de lesiones cutáneas relacionadas con adhesivos médicos. Uberaba, MG, Brasil, 2023

	ERLAM*		
Presencia de MARSI ^†^	Alto riesgo para MARSI ^†^ n ^‡^ (%)	Bajo riesgo para MARSI ^†^ n ^‡^ (%)	Total n ^‡^ (%)
Sí	123 (86,00%)	20 (14,00%)	143 (100,00%)
No	80 (43,00%)	106 (57,00%)	186 (100,00%)
Total n (%)	203 (61,70%)	126 (38,30%)	329 (100,00%)

*ERLAM = Escala de Evaluación del Riesgo para el Desarrollo de Lesiones Cutáneas Relacionadas con Adhesivos Médicos; ^†^MARSI = Lesiones cutáneas relacionadas con adhesivos médicos; ^‡^n = Número de participantes

La confiabilidad interobservadores mostró que 39 (81,5%) ítems de la escala presentaron valores de kappa correspondientes a los mejores índices de concordancia, siendo 30 (62,5%) ítems con concordancia perfecta y nueve (19,0%) con concordancia sustancial. Todos los ítems de la ERLAM alcanzaron niveles de significancia p < 0,05, en su mayoría p=0,001. El nivel de concordancia entre los observadores varió del 75% al 100,0%. El análisis de confiabilidad interobservadores de la ERLAM (Tabla 3) obtuvo un ICC de 0,92, clasificado como excelente^(13)^ y un p < 0,05, lo que demuestra la alta confiabilidad interobservador de la escala.

**Tabla 3-:** Análisis de confiabilidad interobservadores de la Escala de Evaluación del Riesgo para el Desarrollo de Lesiones Cutáneas Relacionadas con Adhesivos Médicos. Uberaba, MG, Brasil, 2023

**Observador**	**Mín.**	**Máx.**	**Media**	**Mediana**	**Desviación estándar**	ICC*	p ^†^
**Obs. 1**	9	22	14,38	14,0	3,36	0,92	< 0,05
**Obs. 2**	9	22	13,81	14,0	3,22		

*ICC = Coeficiente de correlación intraclase; ^†^p = Nivel de significancia

## Discusión

Tras la búsqueda en la literatura, no se identificó la existencia de una escala que clasificara el riesgo de desarrollo de MARSI en adultos hospitalizados. En cuanto a la validez de contenido, la ERLAM presentó valores significativos, próximos a los de las escalas que evalúan el riesgo de lesiones cutáneas, como la Escala de Evaluación de Riesgo para el Desarrollo de Lesiones Derivadas del Posicionamiento Quirúrgico del Paciente (ELPO)^(16)^ y la Escala de evaluación de riesgo de lesión cutánea relacionada con el adhesivo en el sitio de inserción del PICC en pacientes oncológicos^(17)^. En dichas escalas, el índice de validez de contenido (IVC) varió de 0,83^(17)^ a 0,88^(16)^.

En la ERLAM, la diferencia entre las puntuaciones de los grupos clasificados como de alto y bajo riesgo de MARSI fue de más de cuatro puntos en la puntuación total, cercana a la presentada por la ELPO, que obtuvo una diferencia entre grupos con o sin lesión por presión (LP) de casi cinco puntos (p < 0,001)^(16)^. Los análisis de las propiedades métricas de la ERLAM también mostraron excelentes resultados de exactitud, sensibilidad, especificidad y RC. Estos valores se aproximaron a los presentados durante la validación de las demás escalas. La ELPO^(16)^ presentó una exactitud de 0,83, sensibilidad del 88,0%, especificidad del 64,4% y RC de 1,44. Las propiedades métricas de la Braden^(18)^ variaron según el nivel educativo de los profesionales que la emplearon; aunque la sensibilidad de la Braden fue del 100%, la especificidad varió del 64% al 90% entre técnicos y enfermeros, respectivamente. La escala de evaluación de riesgo de lesión cutánea relacionada con el adhesivo en el sitio de inserción del PICC en pacientes oncológicos^(17)^ presentó una exactitud de 0,75, sensibilidad del 80,0% y especificidad del 65,5%, y la escala de evaluación de riesgo para MARSI en bebés y niños de unidad de cuidados intensivos^(19)^ mostró una exactitud de 0,92, sensibilidad del 86,27% y especificidad del 98,76%.

Se destaca que los valores de sensibilidad y especificidad de un instrumento pueden variar, una vez validado, según la población a la que se aplique. Un ejemplo es la escala Braden, que, al ser aplicada en distintos estudios tras su validación, presentó valores de sensibilidad que oscilaron entre el 31,2% y el 95% y de especificidad entre el 16,6% y el 88,2%^(20)^.

La confiabilidad interobservadores de la ERLAM obtuvo resultados excelentes, al igual que los demás instrumentos, tomando como valor de referencia un ICC ≥ 0,75^(14)^. La confiabilidad de la Braden varió según el grado de instrucción del profesional que utilizó la escala (0,83 a 0,94 para auxiliares de enfermería y 0,99 para enfermeros)^(18)^. La ELPO presentó un ICC de 0,99^(16)^, mientras que la escala de evaluación de riesgo de lesión cutánea relacionada con el adhesivo en el sitio de inserción del PICC en pacientes oncológicos^(17)^ tuvo un ICC de 0,97 y la escala de evaluación de riesgo para MARSI en bebés y niños de unidad de cuidados intensivos^(19)^ un ICC de 0,85.

Se identificó como limitación del estudio la ausencia de otra escala de evaluación de riesgo para MARSI en adultos que permitiera realizar la validez concurrente. La ERLAM fue creada y validada en el idioma portugués de Brasil. Por lo tanto, es necesario realizar nuevos estudios para traducir y validar la escala en otros idiomas y, así, posibilitar su utilización en otros países.

La ERLAM podrá contribuir a la práctica de enfermería basada en evidencias, pues reúne de forma objetiva las mejores evidencias científicas sobre los factores de riesgo de MARSI en adultos hospitalizados. Además, al clasificar a esta población según el riesgo de desarrollar dichas lesiones, la escala permitirá a los enfermeros identificar a los pacientes en riesgo de MARSI con mayor rapidez y precisión.

## Conclusión

Mediante los análisis de validez de criterio predictiva y confiabilidad interobservadores, la ERLAM demostró excelente exactitud, sensibilidad y especificidad. Por lo tanto, es un instrumento válido y confiable para identificar el riesgo de ocurrencia de MARSI en adultos hospitalizados.

## Data Availability

Todos los datos generados o analizados durante este estudio están incluidos en este artículo publicado.
